# Untapped potential of 2D charge density wave chalcogenides as negative supercapacitor electrode materials

**DOI:** 10.1039/d2ra00457g

**Published:** 2022-02-23

**Authors:** Mahmoud M. elAttar, Nageh K. Allam

**Affiliations:** Energy Materials Laboratory, School of Sciences and Engineering, The American University in Cairo New Cairo 11835 Egypt nageh.allam@aucegypt.edu

## Abstract

Two-dimensional (2D) materials have opened new avenues for the fabrication of ultrathin, transparent, and flexible functional devices. However, the conventional inorganic graphene analogues are either semiconductors or insulators with low electronic conductivity, hindering their use as supercapacitor electrode materials, which require high conductivity and large surface area. Recently, 2D charge density wave (CDW) materials, such as 2D chalcogenides, have attracted extensive attention as high performance functional nanomaterials in sensors, energy conversion, and spintronic devices. Herein, TaS_2_ is investigated as a potential CDW material for supercapacitors. The quantum capacitance (*C*_Q_) of the different TaS_2_ polymorphs (1T, 2H, and 3R) was estimated using density functional theory calculations for different numbers of TaS_2_ layers and alkali-metal ion (Li, Na and K) intercalants. The results demonstrate the potential of 2H- and 3R-polymorphs as efficient negative electrode materials for supercapacitor devices. The intercalation of K and Na ions in 1T-TaS_2_ led to an increase in the C_Q_ with the intercalation of Li ions resulting in a decrease in the *C*_Q_. In contrast, Li ions were found to be the best intercalant for the 2H-TaS_2_ phase (highest *C*_Q_), while K ion intercalation was the best for the 3R-TaS_2_ phase. Moreover, increasing the number of layers of the1T-TaS_2_ resulted in the highest C_Q_. In contrast, *C*_Q_ increases upon decreasing the number of layers of 2H-TaS_2_. Both 1T-MoS_2_ and 2H-TaS_2_ can be combined to construct a highly performing supercapacitor device as the positive and negative electrodes, respectively.

## Abstract 2

Charge density wave chalcogenides are efficient negative supercapacitor electrode materials with high quantum capacitance.

## Graphical abstract

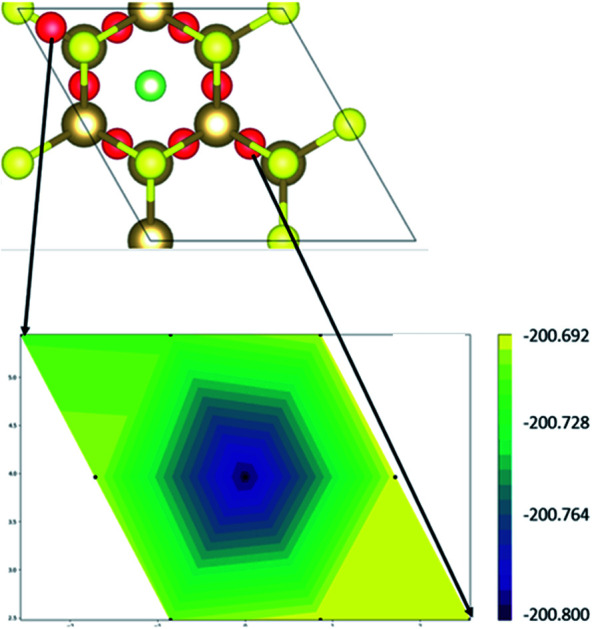

The world energy demand is continuously increasing with time due to the growth of the population that has resulted in more than twice the energy consumption in the last fifty years. Currently, fossil fuels are the major source of energy. However, fossil fuels are associated with severe environmental impacts, such as CO_2_ emissions, which is the major cause of climate change and global warming.^1^ The energy cycle, on the big picture, can be simplified to major three phases, energy generation, storage, and consumption. Those phases can be manipulated *via* high-end inventions to control the consumption efficiency, energy delivery, and side effects.^2^ The second phase can be realized *via* two common ways: energy storage in the form of fuels and electrical (electronic) energy storage systems (ESSs) through batteries and supercapacitors, which are current research subjects.^3,4^ To this end, Li-ion batteries (LiIBs) represent the state of art technology for the ongoing rechargeable energy storage devices.^5,6^ On the other hand, supercapacitors store energy by different mechanisms depending on the active material, namely electrical double layer (EDL) capacitors and pseudocapacitors.^7–9^ The performance of ESSs is usually determined by three factors; energy density, power density, and cyclic stability. Currently, LiIBs offer high energy density, moderate cyclic stability, and moderate-to-low power density. Nevertheless, supercapacitors offer high power density, high cyclic stability, and relatively low energy density.^8^ Therefore, it is important to improve the energy density of supercapacitors, which can be realized by tuning the active electrode material, electrolyte, and separator.^10,11^

Recently, 2D charge density wave (CDW) materials are proved to be very efficient functional materials in many applications such as sensors, spintronics, and energy conversion.^12^ As typical CDW materials, 2D transition metal dichalcogenides (TMDs) exhibit fast charge carrier transport and high charge storage ability. TMDs are layered materials that are stacked together *via* van der Waals (vdW) forces, with the chemical formula XM_2_, where X is a transition element (such as Mo, Ta, W) and M is a chalcogenide element (such as S, Se, Te). TMDs are commonly exist in three phases/polymorphs; 1T (trigonal), 2H (hexagonal), and 3R (rhombohedral).^13–16^ However, little attention was devoted to the investigation of the potential of CDW materials as supercapacitor electrodes. For example, Feng *et al.* reported the use of ultrathin VS_2_ nanosheets in in-plane supercapacitors with high capacitance and outstanding cyclic stability.^14^ As a typical polymorphic TMD, TaS_2_ has been extensively studied for its charge density wave (CDW) and superconductivity characteristics,^17–24^ making it a prompting functional material in energy storage devices. TaS_2_ has also been used to fabricate efficient gas sensors based on its high conductivity.^25^ Therefore, herein, we investigate the potential of TaS_2_ polymorphs as supercapacitor electrode materials using density functional theory (DFT) calculations, which is rarely reported in the literature. To this end, quantum capacitance is modelled and analysed for different number of TaS_2_ layers for the different polymorphs (1T-, 2H-, 3R-TaS_2_). Moreover, the effect of alkali-metal intercalant cations (Li, Na, and K) on the quantum capacitance of the material was investigated and analysed. Density functional theory (DFT) allows for energy calculations of different compounds and structures and estimating the electronic density of states (DOS), which is very useful for extracting quantum related properties form the system under investigation, such as electron diffusivity, transport, conductivity, and quantum capacitance (*C*_Q_). As this work is mainly focusing on supercapacitors, *C*_Q_ is estimated and analysed.^26^*C*_Q_ has been considered a key factor in determining the overall capacitance and storage mechanism in 2D materials, especially graphene,^27^ MoS_2_,^28–30^ doped graphene,^23,31–33^ WS_2_, and TaS_2_.^18,34,35^ In this work a *C*_Q_ estimation is performed based on the simulated DOS and Fermi statistics, where the DOS dependence on the shift in Fermi level (applied gate voltage) is transferred to the Fermi function to extrapolate the calculations for room temperature under different applied gate voltages using eqn (1) and (2):^36–40^1
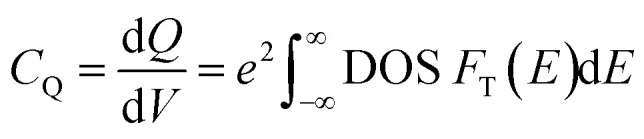
2
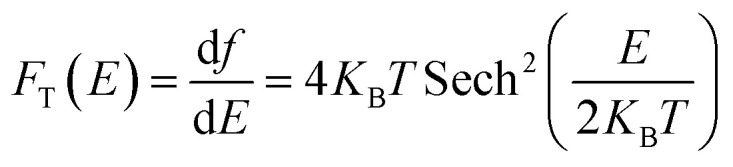
where *F*_T_(*E*) is the Fermi function (*f*) derivative with respect to *E*, *E* is the system energy relative to the Fermi level, which is determined as the applied gate voltage, *T* is the temperature in K (300 K is used as room temperature), and *K*_B_ is the Boltzmann constant. Note that the used assumption of transferring the DOS on the external applied voltage and extending it to room temperature using Fermi statistics is valid if there is no abrupt phase change with temperature and that the external bias voltage is low (in this study the gate voltage is ±0.6 V). Further elaboration regarding these points is provided in the results and discussion section. eqn (3) is used for the formation energy (*E*_f_) calculations, which compares the energy of pristine TMD to the TMD with intercalant:^41^

3 *E*_f_ = (*E*(TaS_2_) − *E*(*X*_c.cc_TaS_2_) − *mE*(*X*)/*n*

where *E*(TaS_2_) is the pristine TaS_2_ total energy, *E*(*X*_c.cc_ − TaS_2_) is alkali metal *X* with concentration c.cc TaS_2_ total energy, *m* number of alkali-metal (intercalant – Li/Na/K) atoms, *E*(*X*) is the isolated alkali metal atom energy, and *n* is the number of TaS_2_ molecules. Three software packages were employed to achieve the best possible efficient workflow and calculations. Vienna *Ab Initio* Simulation Package (VASP) 5.4.4 is used to perform geometry optimization through ionic and electronic relaxations. Electrons charge density and wavefunctions using a self-consistent field run and electronic density of states (DOS). Visualization for Electronic and Structural Analysis (VESTA) is used to build the initial atomic structure, visualizing the relaxed structure, charge distribution, molecular distance, and angles estimation. Python 3.7 is used for DOS reading and processing as well as the quantum capacitance calculation for a defined gate voltage range and increment following the same workflow as per Fig. 1.

**Fig. 1:**
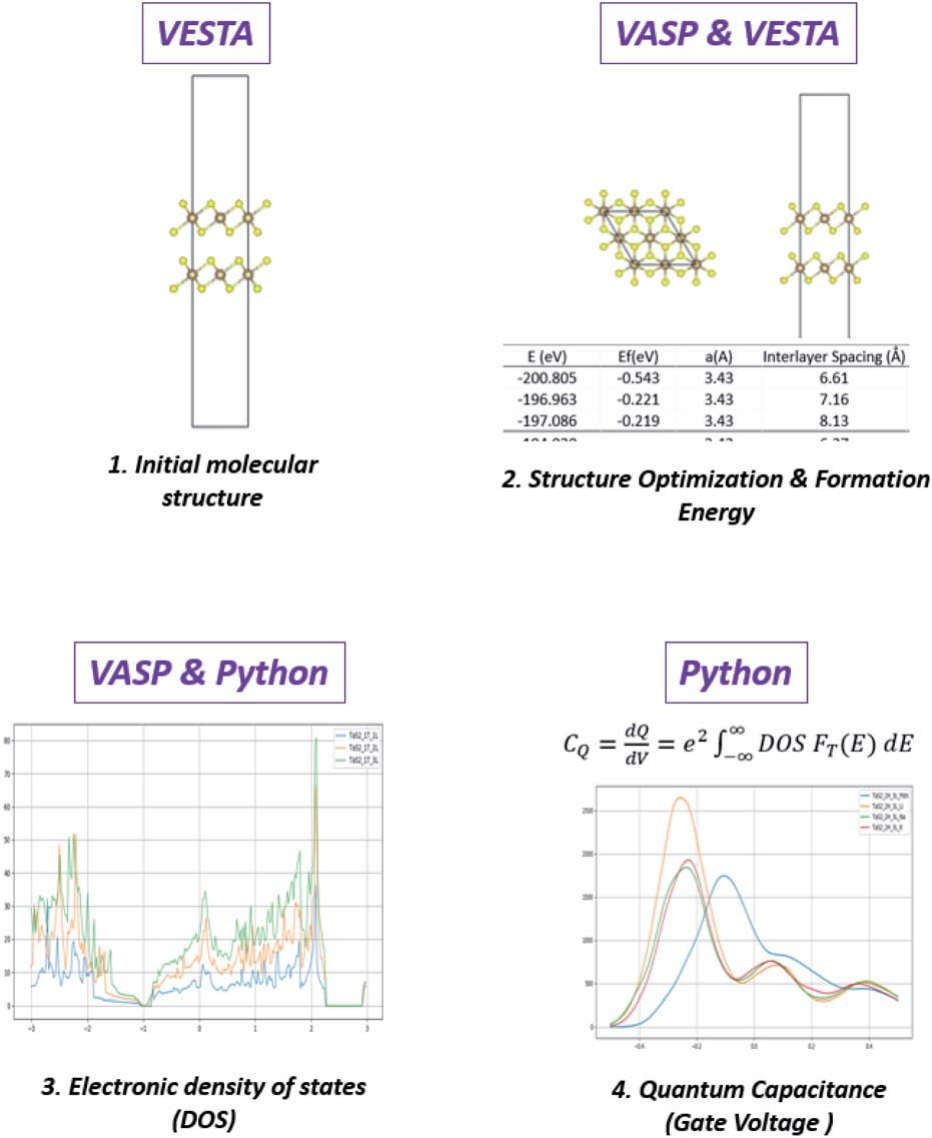
Quantum capacitance investigation workflow.

A 2 × 2 × 1 supercell is used with >30 Å of vacuum equally distributed above and below the layered structure (Fig. 2).

**Fig. 2:**
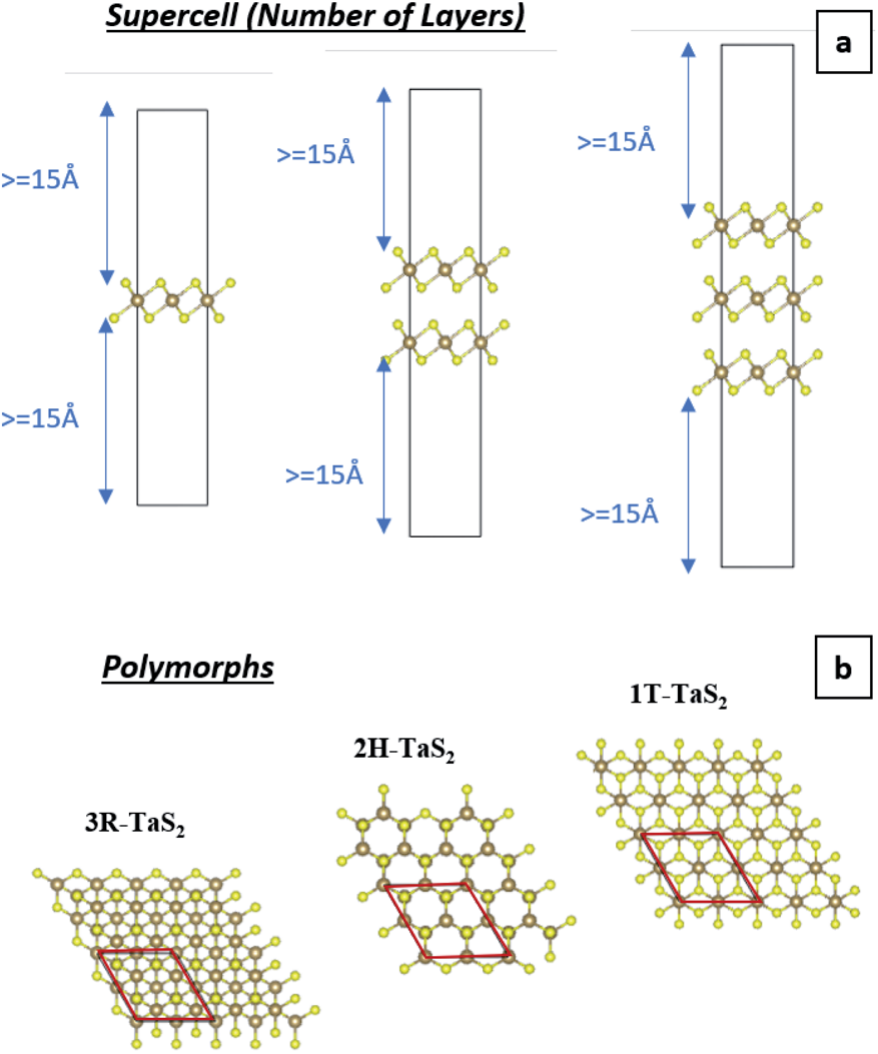
(a) Pristine 1T-TaS_2_ supercells with different number of layers and (b) map-view of the different TaS_2_ polymorphs.

Conjugate gradient algorithm is used for ionic relaxation, cell shape and volume relaxation are enabled, 10^−4^ eV ionic relaxation criteria, 0.2 eV smearing (since we are dealing with metallic system), and vdW enabled using vdW-DF2,^42–45^ which is one of the crucial factors for the simulation of the 2D (vdW) materials. Energy cut-off of 500 eV is used to ensure the convergence of Li s valence orbit during the intercalation calculations. The maximum number of ionic relaxation iterations was 50 iterations. The electronic relaxation conversion threshold was 10^−6^ eV for only the self-consistent field run to generate the final charge density and wave functions. For quantum capacitance estimation, a gate voltage of −0.6 V to +0.6 V was used with step of 1.2 × 10^−2^ V to ensure good sampling of the estimated *C*_Q_ (V). The calculations were performed over four phases. Phase I investigates pristine TaS_2_ in terms of the number of layers (1–3 layers) and different polymorphs (1T, 2H and 3R). Phase II. Prior to the intercalation study it was necessary to determine the most favourable site location for the intercalant. Intercalation site selection is done through starting by the highest symmetry site (green site in Fig. 3a and b) and perturbing to other possible high symmetry sites (red sites in Fig. 3a and b) with full ionic and electronic relaxations are performed to determine the least energy (Fig. 3c). The least energy site is typically the initial guess (green site). Armed with those information phase II operated to investigate for the effect of constant concentration of alkali-metals (AM) intercalation in the 3-layered polymorphs (Fig. 4a). Phase III, performing the same experiments in phase II for 1T-MoS_2_ for the sake of benchmarking the 2H-TaS_2_ results. Phase IV, performing the calculations at different AM concentrations of 16.67%, 33.33%, 50%, and 66.67% in 2H-TaS_2_ with Li and Na intercalants (Fig. 4b–e).

**Fig. 3:**
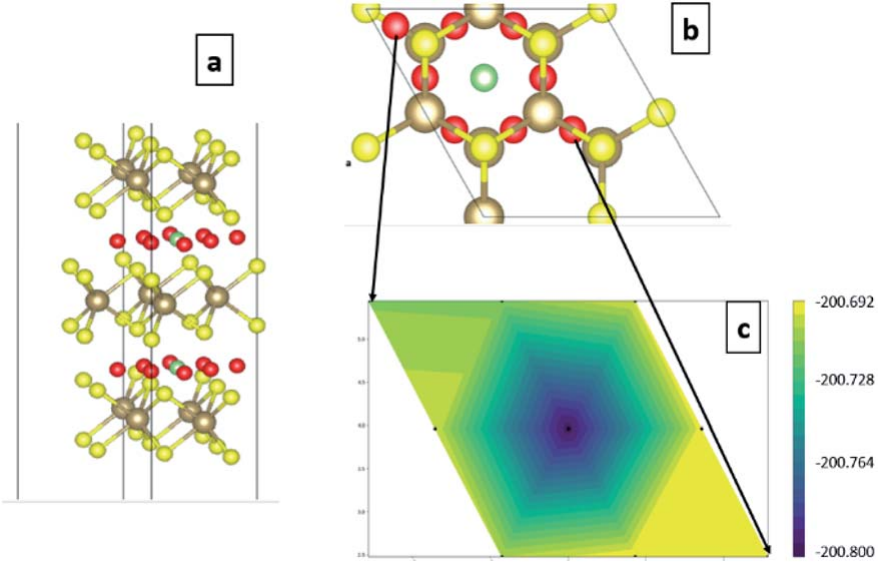
(a) angle-view of the site perturbation (green original and red are the perturbed sites), (b) map-view of the site perturbation, and (c) 3D-contour map for the relaxed energies.

**Fig. 4:**
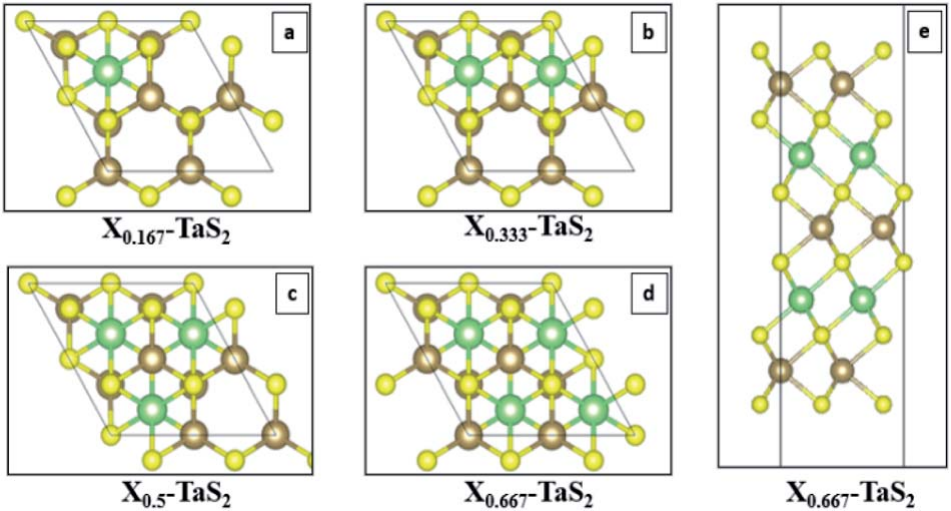
Effect of different concentrations of alkali metal intercalants (a)16.67%, (b) 33.33%, (c) 50%, (d) 66.67% top-view, and (e) 66.67% – side view.

Fig. 5 shows the calculated DOS and the corresponding *C*_Q_ of pristine 1T and 2H TaS_2_ polymorph. Note that pristine 2H-TaS_2_ has a better performance than the 1T-TaS_2_ counterpart in terms of the maximum *C*_Q_ obtained. The maximum calculated *C*_Q_ is 1800 F g^−1^ for the 2H polymorph and 900 F g^−1^ for the 1T polymorph. The 3R-TaS_2_ polymorph showed similar performance to that of the 2H counterpart as shown in Fig. 6c and f. Increasing the number of layers to 2 layers of 1T-TaS_2_ resulted in the highest *C*_Q_. However, in case of 2H-TaS_2_, the *C*_Q_ increases upon decreasing the number of layers. Note that the variation in *C*_Q_ with the number of layers is small (<0.3%). Alkali metal intercalation study using Li^+^, Na^+^, and K^+^ is conducted to determine the quantum capacitance behaviour and the total energy and structural variations associated with the intercalation process, which is crucial to determine the best intercalant ions for the energy storage devices and whether the process is thermodynamically favourable or not. Upon alkali-metal intercalation, Fig. 6, the Fermi level is shifted up for the three polymorphs (1T, 2H, 3R). While the intercalation of K and Na ions in1T-TaS_2_ led to an increase in the *C*_Q_, the intercalation of Li ion resulted in a decrease in the *C*_Q_. Note that the maximum *C*_Q_ occurs very close to 0 V gate voltage, which limits the use of these materials in energy storage systems. In contrast, the 2H phase has an outperforming *C*_Q_ upon Li ion intercalation, reaching 2650 F g^−1^ at gate voltage of −0.26 V, while Na and K intercalation showed *C*_Q_ of 1850 and 1930 F g^−1^, respectively at a gate voltage of −0.23 V with a reasonable enhancement over the pristine counterpart. For 3R case (Fig. 6c and f), K ion intercalation resulted in the best *C*_Q_ of 1950 F g^−1^ at a gate voltage of −0.23 V. A similar performance is observed for Na-intercalation with a *C*_Q_ of 1880 F g^−1^ at the same gate voltage, while Li intercalation resulted in the least *C*_Q_. From energy point of view, pristine 2H- and 3R-TaS_2_ showed lower total energy than their 1T counterpart, indicating the more favourable self-assembly of those two polymorphs. Table 1 lists the calculated formation energy (*E*_f_) for the alkali metal intercalated TaS_2_ polymorphs. The negative *E*_f_ for all different alkali metals intercalated TaS_2_ polymorphs indicates that the formation process is thermodynamically favourable, where Li intercalation.

**Fig. 5:**
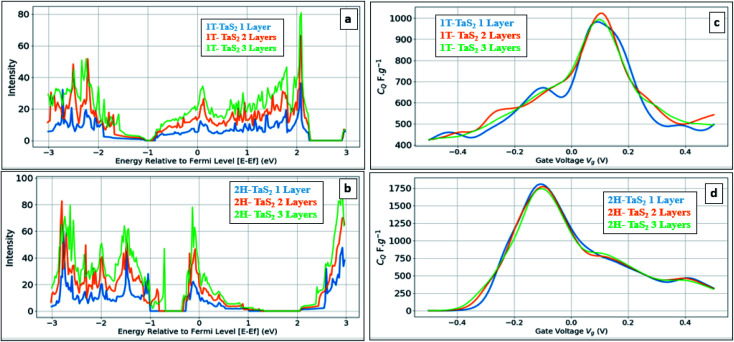
Effect of the number of layers: (a) DOS of pristine 1T-TaS_2_, (b) DOS of pristine 2H-TaS_2_ (c) quantum capacitance *C*_Q_ for 1T-TaS_2_ and (d) quantum capacitance *C*_Q_ for 2H-TaS_2_.

**Fig. 6:**
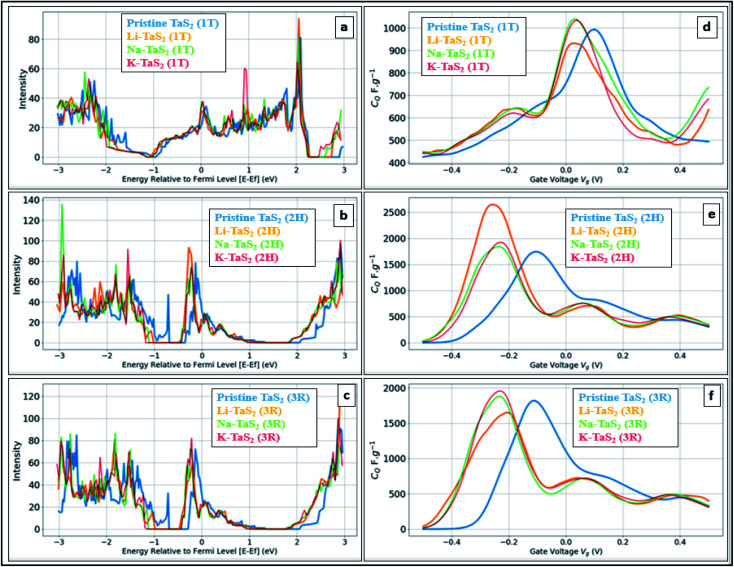
Effect of different intercalants in different polymorphs. (a) DOS of the 1T-TaS_2_ (X = Li, Na, or K), (b) DOS of the 2H-TaS_2_ (X= Li, Na, or K), (c) DOS of the 3R-TaS_2_ (X = Li, Na, or K), (d) *C*_Q_ for 1T-TaS_2_, (e) *C*_Q_ for 2H-TaS_2_, and (f) *C*_Q_ for 3R-TaS_2_ showed the most negative *E*_f_, making it the most favourable intercalant. From molecular structure point of view, intercalating Li leads to ∼4% increase in the interlayer spacing in case of 1T and ∼3.6% in case of 2H and 3R.

**Table 1:** Calculated lattice parameter (*a*), interlayer spacing (*L*), formation energy (*E*_f_), maximum quantum capacitance (*C*_Q_), and gate voltage of the maximum *C*_Q_ (*V*_g_) for pristine and alkali-metal intercalated TaS_2_ polymorphs at constant concentration of the alkali-metal

Compound	Layers	Intercalant	Concentration	*E* (eV)	*E*_f_ (eV)	*a* (Å)	*L* (Å)	*C*_Q_ max (F g^−1^)	*V*_g_ (V)
1T-TaS_2_	1	Pristine	0.000	−64.038	—	3.49	—	980	0.090
	2	Pristine	0.000	−128.914	—	3.49	6.15	1020	0.100
	3	Pristine	0.000	−193.808	—	3.49	6.16	990	0.100
	3	Li	0.167	−199.273	−0.431	3.49	6.41	930	0.030
	3	Na	0.167	−195.192	−0.089	3.50	7.02	1040	0.030
	3	K	0.167	−195.271	−0.084	3.50	8.05	1030	0.040
2H-TaS_2_	1	Pristine	0.000	−64.148	—	3.43	—	1810	−0.100
	2	Pristine	0.000	−129.066	—	3.43	6.39	1780	−0.100
	3	Pristine	0.000	−193.998	—	3.43	6.37	1740	−0.100
	3	Li	0.167	−200.805	−0.543	3.43	6.61	2650	−0.260
	3	Na	0.167	−196.963	−0.221	3.43	7.16	1850	−0.230
	3	K	0.167	−197.086	−0.219	3.43	8.13	1930	−0.230
3R-TaS_2_	3	Pristine	0.000	−194.020	—	3.43	6.37	1820	−0.110
	3	Li	0.167	−200.905	−0.549	3.43	6.62	1640	−0.200
	3	Na	0.167	−196.968	−0.219	3.43	7.23	1880	−0.230
	3	K	0.167	−197.066	−0.216	3.43	8.25	1950	−0.230
1T-MoS_2_	3	Pristine	0.000	−163.480	—	3.34	6.18	2361	0.270
	3	Li	0.167	−173.272	−0.792	3.39	6.44	1230	0.420
	3	Na	0.167	−169.322	−0.460	3.39	7.05	1300	0.420
	3	K	0.167	−169.587	−0.471	3.39	8.04	1100	0.390

On the other hand, Na intercalation resulted in ∼13% increase in cell size and K intercalation showed 28% increase. Therefore, in general, 2H and 3R polymorphs are better performing in terms of quantum capacitance upon alkali metal ion intercalation, which qualify them to be used as negative electrodes in energy storage devices. Upon increasing the alkali-metal ion concentration, the calculations showed the *C*_Q_ to decrease, see Fig. 7. For example, a drop of 55% for Li and 73% for Na was observed upon increasing the concentration from 16.67% to 66.67%. On other hand, increasing the alkali metal leads to a shift of the gate voltage of the maximum quantum capacitance towards the negative side, reaching −0.36 V in case of Na and −0.41 in case of Li. A compromise between the energy density (which favours the high quantum capacitance) and power density (which favours the high gate voltage) should be made based on the energy storage device. From formation energy point of view, high alkali metal (AM) concentration is thermodynamically favoured up to the ratio of 8AM : 12TMDs. From molecular structure point of view, a negligible variation in interlayer spacing is observed for all concentrations with slight increase in lattice parameter *a* (Å) with maximum variation of 1% in case of Na 0.667 concentration, see Table 2. As the calculations showed the suitability of alkali-metal ion intercalated TaS_2_ for use as negative electrodes, we have investigated the metallic 1T-MoS_2_ phase under the same framework, see Fig. 8. Note that 1T-MoS_2_ is better behaving as a positive electrode. Therefore, both1T-MoS_2_ and 2H-TaS_2_ can be combined to construct a supercapacitor device as the positive and negative electrodes, respectively.

**Fig. 7:**
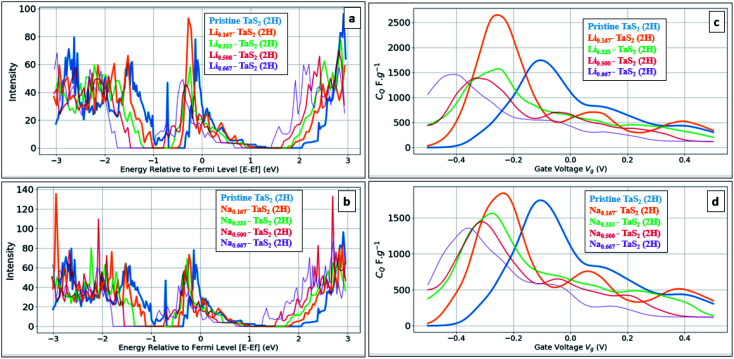
Comparison of different alkali metal concentration for 2H-TaS_2_ (2H). (a) DOS for Li_c_-TaS_2_, *C* = 0.167, 0.333, 0.500, and 0.667, and (b) DOS for Na_c_-TaS_2_, *C* = 0.167, 0.333, 0.500, and 0.667, (c) quantum capacitance *C*_Q_ for Li_c_-TaS_2_, *C* = 0.167, 0.333, 0.500, and 0.667, and (d) quantum capacitance *C*_Q_ for Na_c_-TaS_2_, *C* = 0.167, 0.333, 0.500, and 0.667.

**Table 2:** Calculated lattice parameter (*a*), interlayer spacing (*L*), formation energy (*E*_f_), maximum quantum capacitance (*C*_Q_), and gate voltage of the maximum *C*_Q_ (*V*_g_) for pristine and alkali-metal intercalated TaS_2_ polymorphs at different concentrations of the alkali-metal

Compound	Intercalant	Concentration	*E* (eV)	*E*_f_ (eV)	*a* (Å)	*L* (Å)	*C*_Q_ max (F g^−1^)	*V*_g_ (V)
2H-TaS_2_	Pristine	0.000	−193.998	—	3.43	6.37	1740	−0.100
	Li	0.167	−200.805	−0.543	3.43	6.61	2650	−0.260
	Li	0.333	−207.043	−1.038	3.44	6.62	1750	−0.250
	Li	0.500	−212.694	−1.485	3.44	6.62	1390	−0.330
	Li	0.667	−217.740	−1.881	3.44	6.62	1460	−0.410
	Na	0.167	−196.963	−0.221	3.43	7.16	1850	−0.230
	Na	0.333	−199.388	−0.423	3.46	7.25	1560	−0.280
	Na	0.500	−200.843	−0.544	3.46	7.21	1450	−0.310
	Na	0.667	−201.421	−0.592	3.48	7.17	1360	−0.360

**Fig. 8:**
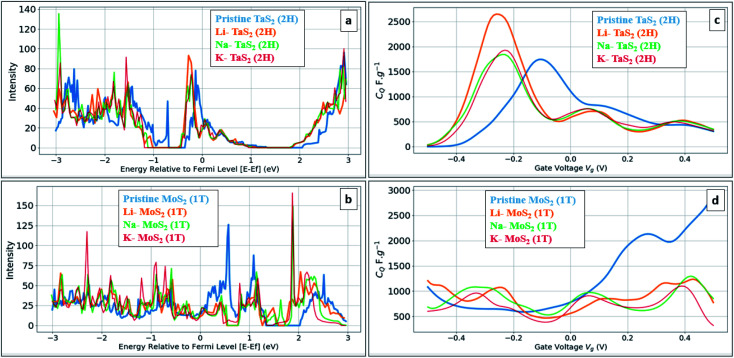
Comparison between the outperforming polytypes of TaS_2_ and MoS_2_: (a) DOS of 2H-TaS_2_ with different alkali-metal intercalation (b) DOS of 1T-MoS_2_ with different alkali-metal intercalation (c) Quantum Capacitance *C*_Q_ for 2H-TaS_2_ with different alkali-metal intercalation, and (d) Quantum Capacitance *C*_Q_ for of 1T-MoS_2_ with different alkali-metal intercalation.

## Conclusion

An in-depth quantum capacitance first-principles investigation is demonstrated for TaS_2_ with different number of layers, polymorphs, and alkali metal intercalants. The study shows that TaS_2_ is promising material for low bias electrical energy storage systems (ESS) such as supercapacitors. The 2H and 3R TaS_2_ phases are more stable and better performing (higher quantum capacitance) than the 1T phase. While Li ion was found to be the best intercalant for the 2H-TaS_2_ phase (highest *C*_Q_), K ion intercalation was the best for the 3R-TaS_2_ phase. Increasing the alkali metal ion concentration resulted in a decrease in *C*_Q_ the of the 2H-TaS_2_ phase with a shift in the gate voltage of the peak *C*_Q_ to the more negative side. Our investigation also showed the promise of combining the 2H-TaS_2_ as a negative electrode with the 1T-MoS_2_ as a positive electrode to construct a highly performing supercapacitor device. In this regard, a threshold needs to be made to ensure high energy density, which favours high quantum capacitance, and high power density, which favours high gate voltage.

## Author contributions

Mahmoud M. el Attar: methodology, formal analysis, investigation, writing original draft. Nageh K. Allam: conceptualization, formal analysis, writing – review & editing, project administration, funding acquisition, supervision.

## Conflicts of interest

The authors declare that they have no known competing financial interests or personal relationships that could have appeared to influence the work reported in this paper.

## Supplementary Material
